# Management of Glaucoma in an Adult Presentation of Sturge-Weber Syndrome

**DOI:** 10.7759/cureus.23699

**Published:** 2022-03-31

**Authors:** Ali Al-Smair, Hammam Rababaa, Ahmad Saadeh, Ahmad Al-Ali

**Affiliations:** 1 Department of Radiology, Medray International Radiology Center, Amman, JOR; 2 Faculty of Medicine, The University of Jordan, Amman, JOR; 3 Department of Radiology, Jordan Ministry of Health, Amman, JOR

**Keywords:** choroidal hemangioma, case report, glaucoma, treatment failure, sturge-weber syndrome

## Abstract

Sturge-Weber syndrome (SWS) is a rare neurocutaneous syndrome. It is described by the presence of leptomeningeal angiomas, ocular involvement such as choroidal hemangioma and glaucoma, and port-wine stain over the face. Management of SWS-associated ocular complications is challenging and needs regular follow-ups. Herein, we present a case of a 28-year-old male patient who underwent glaucoma surgery but did not adhere to regular follow-ups and later presented with left-sided exophthalmos and eye pain. Management with medical treatment (latanoprost) was effective in the short term, but regular follow-ups are crucial to prevent further progression due to high failure rates.

## Introduction

Sturge-Weber syndrome (SWS), also known as encephalotrigeminal angiomatosis, is a neurocutaneous disorder. It is a rare, sporadic, congenital disorder, which affects one newborn in approximately 50,000 live births. It is characterized by a port-wine stain that affects the skin in the ophthalmic division of the trigeminal nerve and is associated with venous-capillary malformations of the leptomeninges and the eye [1]. These vascular malformations are associated with specific neurologic and ocular manifestations such as headaches, seizures, hemiplegia, and intellectual disability. Choroidal hemangiomas occur in 20-70% of patients with SWS, which may cause vision loss from choroidal thickening or retinal detachment. Also, glaucoma occurs in 30-70% of SWS patients, usually unilateral and often diagnosed in infancy, though it can develop later [2]. The diagnosis of SWS is easily performed when the classical clinical signs are present; however, the management of SWS is difficult because of the lack of consensus guidelines for the care, surveillance, and follow-up. This limitation may be accentuated in SWS patients with cognitive impairment, by the emotional and psychological burdens on the patients, and in developing countries, where such regular follow-up is hard to achieve [3]. Herein, we present a case of a 28-year-old male patient who was born with a port-wine stain but did not adhere to the clinical follow-ups, who presented with left-sided exophthalmos and eye pain. To the best of our knowledge, this is the first case of an SWS-related complication to be reported in Jordan, a developing, middle-income country.

## Case presentation

A 28-year-old male presented to the emergency department complaining of left eye bulging for two months and a two-day history of left eye pain. The patient was born with a port-wine stain on the left side of his face. He was diagnosed with glaucoma, which was treated surgically by a drainage device in his early 20s, but he had no follow-up since that time. He had no history of seizures, weakness, or loss of consciousness. On examination, there was bulging in the left eye, redness, pain (no increase in pain upon eye movement), and mid-dilated pupil. There was no fever or eye drainage apart from tearing. There was no recent history of eye trauma. The right eye was normal-looking, non-painful, and had no eye drainage. Visual acuity was examined. The left eye showed a decrease in visual acuity to 4/6. Visual field examination showed a decrease in the peripheral visual fields of the left eye, while it was completely normal on the right eye. On ophthalmoscopy, the left eye showed an increased cup to disc ratio (0.7) and normal cup to disc ratio in the right eye. On tonometry, an elevation in the intraocular pressure (IOP) was seen in the left eye that measured 35 mmHg, while in the right eye, it was 18 mmHg. His cognitive function was normal. Regular blood workups were within normal limits, including the white blood cell count. The patient was managed in the emergency room with timolol, acetazolamide, and pilocarpine to lower his IOP. After stabilizing the patient, he was started on prostaglandin analog (latanoprost) for the management of glaucoma, which helped reduce IOP until the ophthalmology appointment to discuss surgical options for definitive treatment.

**Figure 1:**
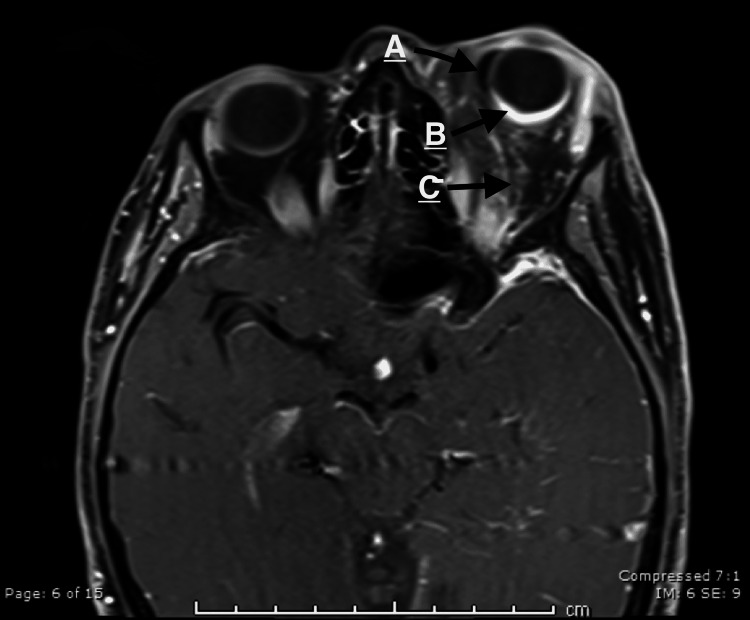
(A) A glaucoma drainage device. (B) An enhancement at the posterior aspect of the left eye indicating choroidal angiomatosis. (C) A heterogeneous retro-orbital fat with exophthalmos.

Magnetic resonance imaging (MRI) of the head revealed bulged and enlarged left eye globe, abundant retro-orbital heterogenous fat, and left optic nerve atrophy at the orbital apex. Left-sided glaucoma drainage device was noted at the medial aspect of the left eye globe. Also, MRI showed a thickened and enhanced posterior aspect of the left eye globe, which suggests left choroidal hemangioma (Figure 1).

**Figure 2:**
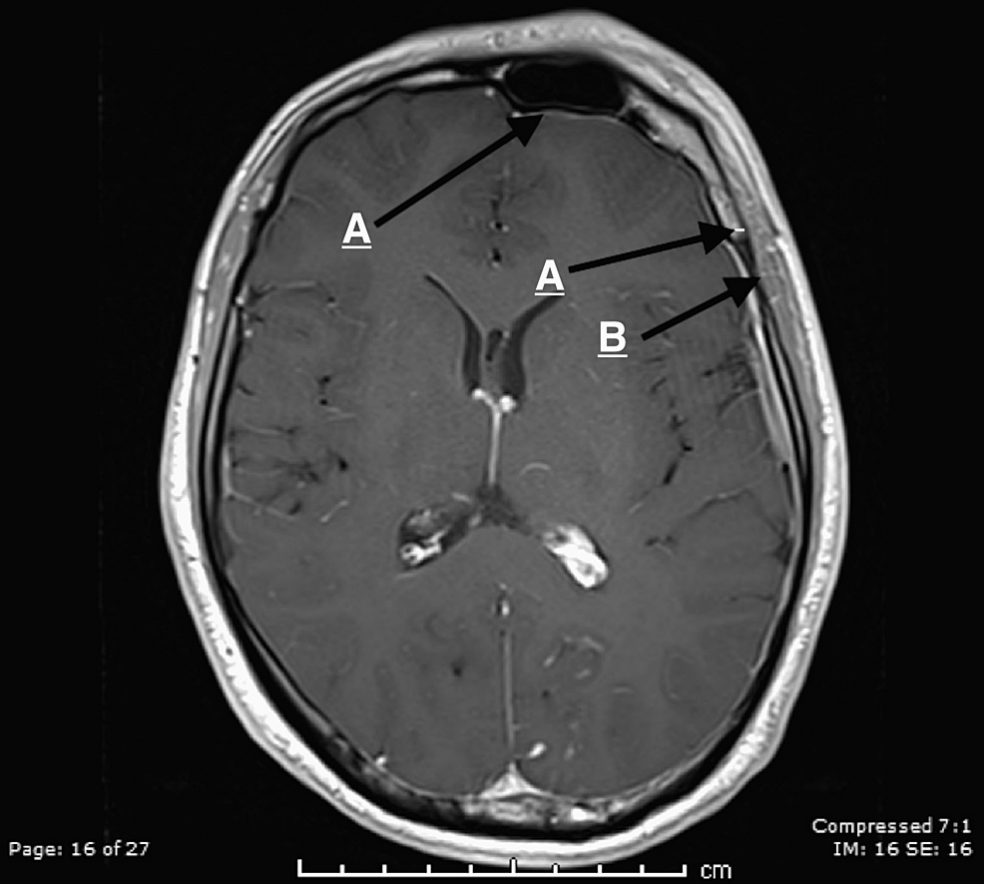
(A) Mild left cerebral atrophy. (B) Left calvarial thickening.

Mild atrophy of the left cerebral and cerebellar hemispheres with left frontal calvarial thickening was noted (Figure 2).

**Figure 3:**
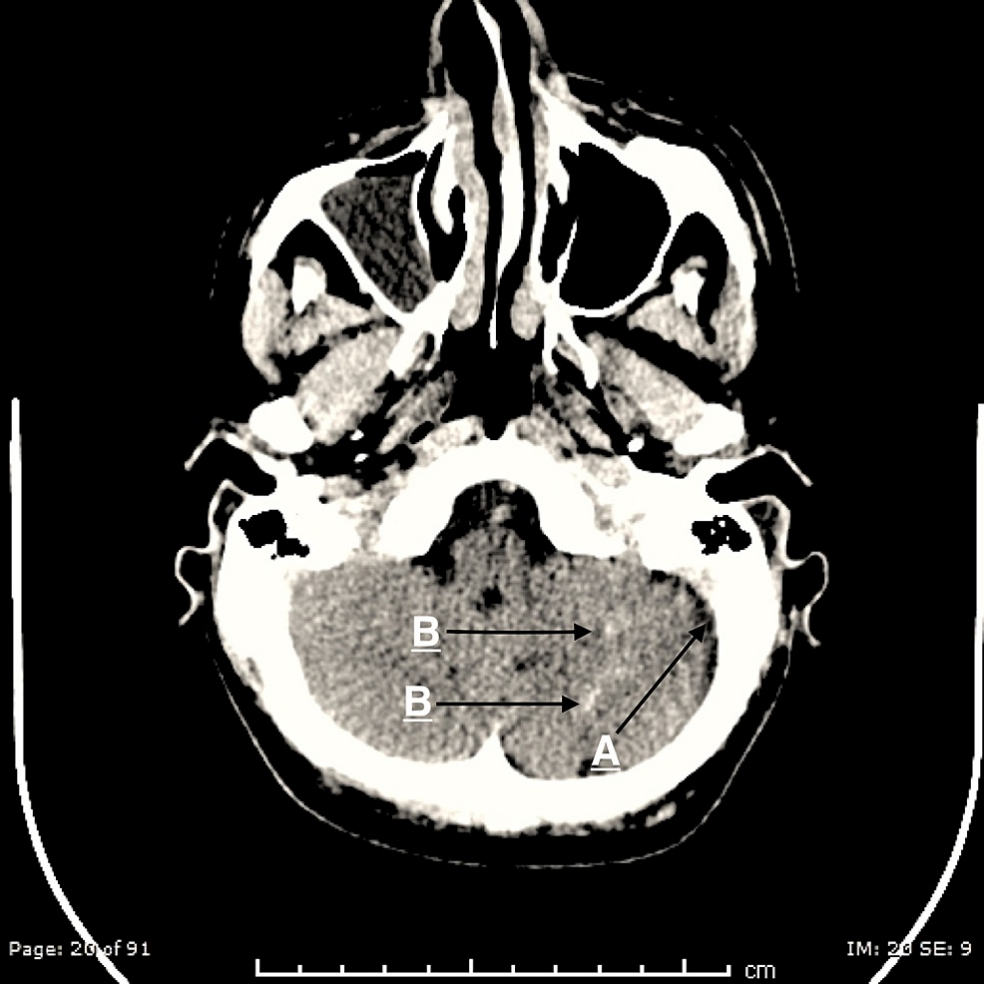
(A) Mild left cerebellar atrophy. (B) Left cerebellar curvilinear densities suggesting left cerebellar pial calcifications.

Curvilinear enhancement of the left cerebellar hemisphere subarachnoid spaces is noted, which indicates pial angiomatosis (Figure 3).

**Figure 4:**
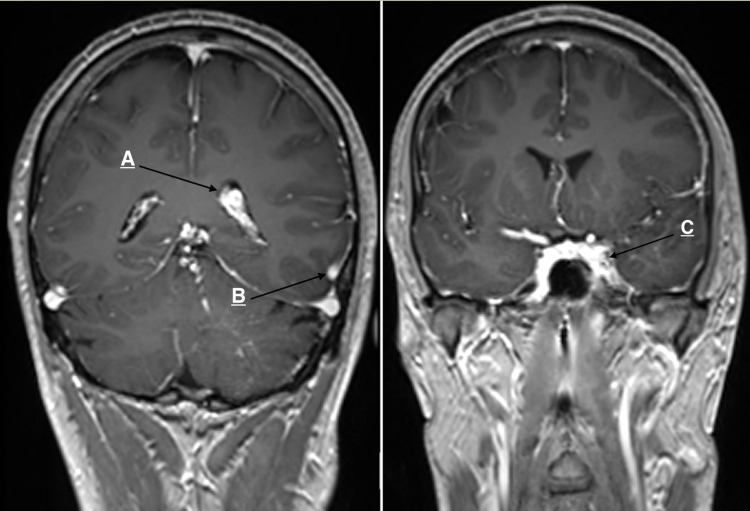
(A) Enlarged left choroid plexus. (B) Dilated left vein of Labbé. (C) Dilated cavernous sinus.

Observation of a dilated left vein of Labbé and multiple enhancing vessels draining via a single larger vein into the deep veins, with choroid plexus enlargement, suggested multiple venous angiomas (Figure 4).

Based on the clinical and radiological findings, the patient was diagnosed with SWS. A follow-up with ophthalmology, neurology, and plastic surgery clinics was scheduled for the patient, but unfortunately, he did not attend any clinic.

## Discussion

SWS is a rare neurocutaneous syndrome, caused by a mutation in the GNAQ gene that is involved in blood vessel development [1]. Sturge described it in 1879 as the association of facial port-wine stain, glaucoma, and focal seizures. Later, Weber set out the radiological features, i.e., pial angiomatosis and brain involvement [4]. Although SWS is described by the presence of ocular abnormalities, facial angiomatosis, and leptomeningeal hemangioma, these clinical findings may vary, and different types of SWS are recognized based on these variable neural and ocular signs and symptoms, along with neuroimaging and systemic findings [2].

Glaucoma is one of the most frequent ocular manifestations in SWS (30-70% of patients). It is usually open-angle, associated with other findings (e.g., buphthalmos), and has a bimodal peak: an early-onset (congenital), which is a more common form, and a later-onset form during childhood and adolescence. Moreover, choroidal hemangiomas are present in 20-70% of SWS patients, and this can increase the risk of glaucoma development. Although choroidal hemangiomas may be asymptomatic, severe forms may lead to visual impairment through exudative retinal detachment and macular edema [2,3].

To halt glaucoma progression and the impending visual field damage, management of these ocular complications in SWS is mandatory. However, SWS-associated glaucoma is a challenging disease due to its early development and poor response to standard medical and surgical treatment [2]. In our case, the patient was diagnosed with glaucoma in his early 20s and he was treated surgically with a valve implant. Since he had no clinical follow-up till this presentation, his left eye IOP returns to elevate, which indicates the failure of the surgery, like other studies that showed unfavorable outcomes in these patients [3,5].

However, Ong et al. (2003) showed that using latanoprost eye drops as adjunctive therapy was effective in controlling SWS-associated glaucoma in seven of 14 patients at one year of follow-up [6]. In line with this, our patient demonstrated a decrease in IOP with latanoprost treatment.

Because of the progressive nature of these ocular complications and the high failure rates in standard treatments, it is crucial to plan for regular follow-up visits with complete ophthalmic examinations in SWS patients. However, many factors could contribute to poor adherence to follow-up in these patients, including the lack of consensus guidelines for the care, surveillance, and follow-up; the cognitive impairment that they may have; and the emotional and psychological burdens on them [2,3]. This challenge may be compounded in developing countries (e.g., Jordan, in our case), as evidenced by the low rates of follow-up in many low and middle-income countries [7].

## Conclusions

Our clinical case demonstrates the radiologic findings and some of the drawbacks in the management of SWS, raises awareness of radiologists for the subtle findings in neuroimaging, and calls clinicians, especially ophthalmologists, to develop measures that can improve the outcomes and follow-up adherence in these patients.
